# Proposing new path-planning metrics for operating rovers on Mars

**DOI:** 10.1038/s41598-023-49144-8

**Published:** 2023-12-14

**Authors:** Pablo Muñoz, Paolo Bellutta, Maria D. R-Moreno

**Affiliations:** 1 Universidad de Alcalá. EPS. ISG Group, 28871 Alcalá de Henares, Madrid Spain; 2 Jet Propulsion Lab (JPL-NASA), Pasadena, CA 4800 USA; 3 TNO, IAS, The Hague, The Netherlands

**Keywords:** Astronomy and planetary science, Engineering

## Abstract

The on-ground operation of Mars rovers is a complex task that requires comprehensive planning in which path planning plays a fundamental role. The selection of paths has to be carefully chosen considering the scientific objectives, terrain, energy, and safety. In this regard, operators are assisted by path-planning algorithms that generate candidate paths based on cost functions. Distance traveled has always been considered one of the primary criteria when comparing paths. Other metrics such as the run-time to generate the solution or the number of expanded nodes are common measures considered in the literature. However, we want to analyze if those metrics provide useful information in challenging and partially known terrain. In this paper, we will review those metrics using two-path planning algorithms on real Mars maps. Based on our experience operating Mars rovers, we propose new metrics for assessing paths in real-world applications.

## Introduction

Operating a very expensive vehicle (such as a Mars rover) in a partially known environment with a high expected scientific impact is a complex and stressful task^1^. The loss of mobility in a rover mission can result in a significant or complete loss of scientific return. It is therefore imperative to maintain mobility safely by careful path planning around obstacles and hazards, under the constraints such as available energy and slope limitations.

While much is still unknown about operating a robotic mission on another planet, several instruments and tools have been brought to the science community in the last two decades.

Orbiters and previous surface missions (two of the most recent rover missions, Mars Exploration Rovers (MER) in 2004 and Mars Science Laboratory (MSL) in 2012) provided more and more detailed information about Mars’ terrain terramechanics properties and environmental conditions. Each vehicle includes digital still cameras that capture multispectral high-resolution 3D images, allowing a detailed analysis of the surface morphology and composition. Other tools have been developed to show terrain slope, surface roughness, and distance to potential obstacles. Machine vision-based terrain classifiers have been developed to provide automated terrain evaluation from orbital imagery^2,3^. These classifiers are computer algorithms designed to analyze and interpret orbital imagery, in our case planetary surfaces. These classifiers aim to automatically identify and categorize different types of terrain or features within the images, making it possible to extract valuable information about the surface without manual intervention.

On-ground operators also have access to simulators and engineering models to evaluate the possible outcome of maneuvers before being deployed on Mars. All these tools are mostly used to define tactical (daily) activities but on a strategic level operators may be called to define activities that stretch over many months of activities. Tools have been developed for both MER and MSL missions to quantify terrain traversability which can be used to provide safe and optimized routes between targets.

In this paper, we will focus on path-planning algorithms. Path planning is a widely researched problem that aims to find an optimal (or close-to-optimal) path, avoiding known obstacles^4^. Typically, path planning algorithms used for rovers’ operation use 2.5D maps to represent the traversable regions on Mars. The 2.5 maps are represented as cost maps using 2D fixed-grid cell maps. Each path generated by the path planning algorithm is defined by the driving steps plus a set of metrics to indicate the quality of the path. Such metrics entail the assessment of the distance traveled, search run-time, and number of expanded nodes. These metrics are commonly used among path planning works^4–7^, and from them other metrics can be derived, such as the heading changes or energy consumption^8–10^. As in other research areas, these metrics are also used to compare algorithms to determine if a new development outperforms previous solutions. Although these metrics are strongly established in the research community, we wonder if they can be used as a guide to decide which algorithm to use for autonomous rovers on Mars or any planetary surface. As we will show in the paper, there are some aspects of designing and operating a mission that are not fully captured in these “classic” metrics.

For this reason, in this paper, we will focus on discussing the advantages and drawbacks of such metrics. Based on the decisions made on the actual paths followed by Opportunity and Curiosity rovers and the experience of a rover driver, we propose new metrics such as ease of implementation by using the Drive Vector (DV) concept or mission duration to capture most of the criteria used by rover drivers during daily operation. To exemplify the metrics, we have used the paths generated by two algorithms: an adapted version of Field D*^11^, used at JPL to generate the paths for MER and MSL missions, and 3Dana^7^. The authors want to emphasize that the purpose of the paper is not to compare the algorithms but to use them to assess the proposed metrics comparing them with the “classic” path planning metrics.

In the following section, we will introduce a concise survey on path-planning algorithms. Subsequently, we will delve into the operational procedures employed by rover drivers when maneuvering rovers on Mars. To illustrate this, we will provide an example of a specific Sol from the Opportunity rover’s mission. We will then outline the common approach to terrain discretization in path planning and offer insights into the two algorithms discussed in this paper: Field D*^5,11^ and 3Dana^7^. Moving into the experimental section, we will introduce both classical and our proposal on new path planning metrics. These metrics will be applied to analyze and assess two sample paths that were followed by the Opportunity and Curiosity rovers, respectively. Lastly, we will conclude with a discussion of our findings and a summary of the paper’s key takeaways in the conclusion section.

## Related works

The classical approximation in a 2D environment envisions path planning as a search problem abstracted by a search graph. One of the oldest and commonly used algorithms is the Dijkstra algorithm^12^ that finds the optimal path expanding all the search space. Another very well-known algorithm is A*^13^. It is based on heuristics (i.e. Euclidean or Manhattan distances) so it significantly reduces the search space. However, A* generates synthetical paths with zig-zag patterns (generally, values of $\pi /4$ considering 8 neighboring edges) as the movements are constrained to the adjacent nodes. To eliminate this, A* with postprocessing (A*PS)^14^ smoothes the path obtained by A* in a post-processing step, reducing the total length. We can also mention Theta*^15^ and S-Theta*^16^ that eliminate this problem thanks to the inclusion of a Line of Sight procedure during the search process. This procedure checks if there are no obstacles between two adjacent nodes, allowing movement between no adjacent nodes.

Otherwise, both algorithms work like A*. Another way of alleviating this problem is the approximation of Field D*^5^ that uses linear interpolation to efficiently produce low-cost paths. Other approaches consider the terrain information during the search, such as the works of Sun and Reif^10^ and Choi et al.^17^ that propose path planning algorithms based on the energy consumption crossing a 2.5D elevation map representation. In a similar direction, Garcia et al.^18^ present an integrated 3D path planning architecture composed of two stages: acquisition and map generation. In the acquisition phase, the rover obtains the terrain elevation. Subsequently, a fuzzy engine produces a 2D navigational map associating a cost for each (*x,y*) pair, represented by a continuous value from 0 to 1. Then, a path planning module generates the path based on a modified version of A*.

There are other approximations based on Metaheuristics^19,20^ or randomized sampling-based methods such as Rapidly exploring Random Tree (RRT)^21^ or Probabilistic Roadmap (PRM)^22^ that can also solve the problem from a different perspective. Other works try to improve A* by combining it with different search strategies^23,24^.

There are many proposals in literature to solve this problem but here we focus only on those that use grid-based representations, which are commonly used for real applications. The reason grid-based planners are used for real missions is due to the fact the onboard flight software uses grid-based software which allows for predictable resource usage. Moreover, previous algorithms have in common the metrics used to evaluate and compare them. Typically, the distance traveled or the length of the solution, the run-time to generate the solution, and the number of expanded nodes during the search phase. Other authors propose the energy consumption for turns and accelerations of different paths at different velocities as a comparison metric^9^. Muñoz et al.^16^ also propose heading changes as another comparison metric since the least-cost path may imply large heading changes, that may be associated with higher energy usage in some mobile platforms. We could see that some authors start including also this metric in their comparison^23,24^.

## Operating rovers on Mars

Currently, NASA/JPL Mars rover operators use a combination of orbital and surface imagery to analyze the terrain topography and composition to determine where it is safe to drive the vehicle and which routes to take. While this can be done on a day-to-day basis, whenever operators need to decide on a strategic route (a path that is defined using orbital imagery and relates to multi-Sol activity(ies), possibly for kilometers/years), then some automation in this process is necessary.

From stereo image pairs a Digital Terrain Model (DTM) and terrain slope are computed on-ground. In addition, using HiRISE http://www.uahirise.org/ images, a machine vision terrain classifier is used to locate the most likely terrain hazards such as sandy areas or sand dunes or obstacles such as rocks or scarps. Knowledge of the vehicle mobility performance allows quantifying terrain traversability at each location.

**Figure 1:**
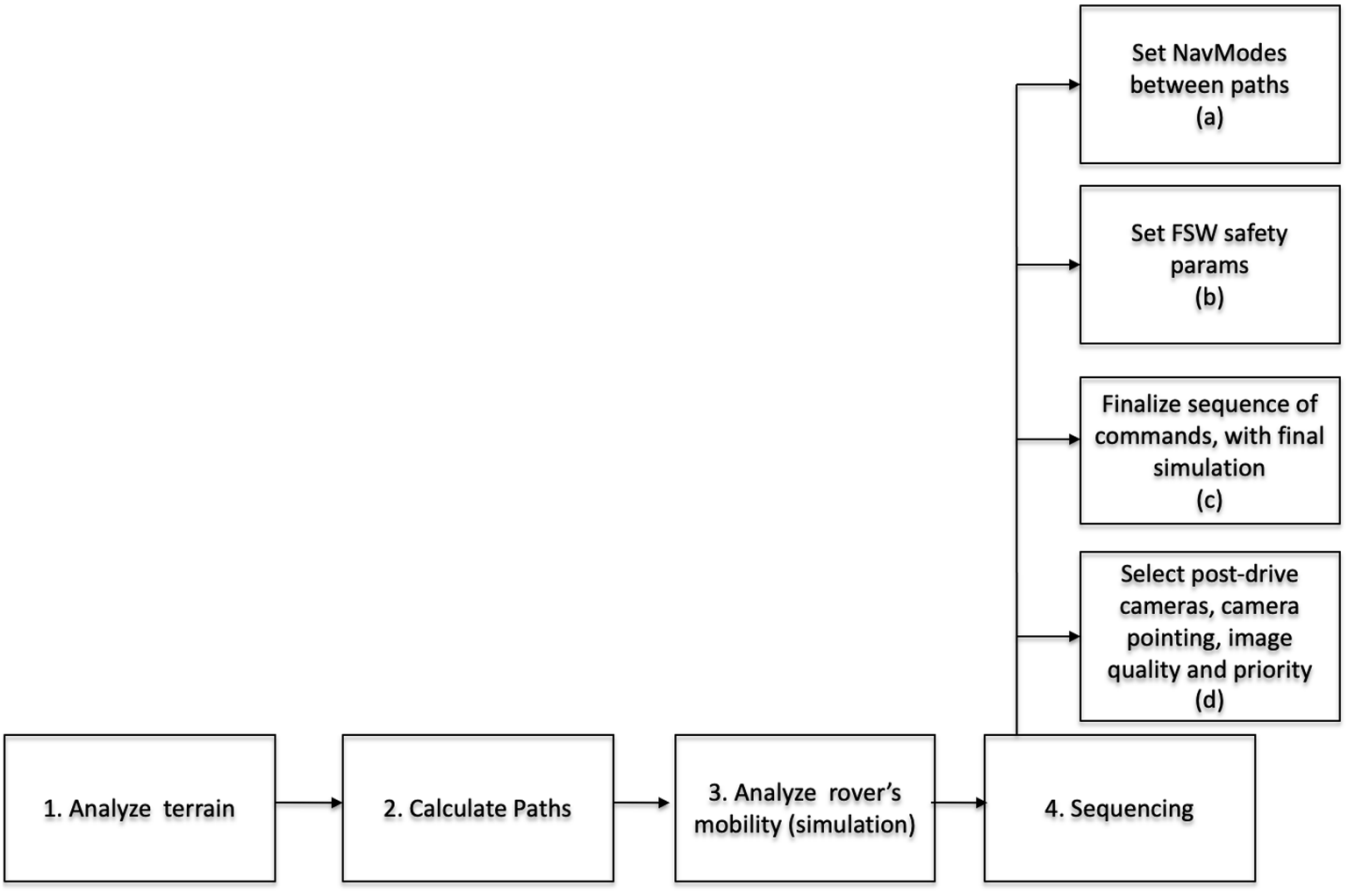
General steps for sending commands to the Mars rovers.

Figure 1 represents the general sequence of actions that drivers should follow to successfully complete the activity of deciding the commands that should be sent to the rover through the Deep Space Network (DSN) based on the downloaded images of the previous day. Next, those activities are explained.

1. **Analyze terrain** topography and composition: the drivers must avoid areas where slopes are higher than what the vehicle can traverse, such as 25$^o$ and above on bedrock or 12$^o$ and above on well-sorted sand and rocks, or scarps higher than what the rover can climb. That information comes from the machine vision-based terrain classifiers. Traversable terrain is typically classified into five groups of increasing difficulty:
  No significant slopes or vehicle hazards such as rocks and scarps.
  No significant slopes, some rover hazards/obstacles but with ample room to maneuver using onboard Autonomous Navigation (AN).
  No significant rover hazards/obstacles, terrain has a moderate slope which requires the use of onboard Visual Odometry (VO).
  Moderate slopes and rover hazards/obstacles are present which require the use of both, AN and VO.
  Terrain where slopes are too steep and/or there is too little room to maneuver around hazards or obstacles.
  Each group of terrain traversability uses a different software configuration of increasing complexity. As a result, while the vehicle motion is always a set value, the corresponding effective drive rates decrease as the terrain difficulty increases.
2. **Calculate paths** between Waypoints (WPs): using an automated process developed for the MSL landing site selection, the information of the terrain is calculated automatically by the available SW. This is translated into a cost map, merging different terrain characteristics such as slopes, rocks, etc into the map with different cost values. This information is taken into consideration when calculating the path to avoid risk areas (that is, areas with higher costs are avoided since we want to minimize the path). The WPs are introduced by scientists or operators based on the mission needs.
  Once a cost map is computed, path planning algorithms are called to find optimal solutions to safely drive the rover through different WPs (set by the operators based on scientific goals). When the path is generated, surrounding hazards and obstacles need to be considered in the context in which they are located. For example, a path uphill of a large rock field should be considered more hazardous than a path downhill from it as the rover could slide toward and crash into these hazards. Terrain type also plays a major role in determining preferred paths. Bedrock can provide good traction but can also damage the vehicle wheels. Narrow passages should also be avoided to ensure there is sufficient room to maneuver in case the intended drive path is not successful. While considerable data has been gathered about terramechanics properties of the Martian soil, much is still unknown.
  We convert the path once it is generated by the path planning algorithm as a series of vectors, then compute its performance by counting the cost of traverse (sum of the cost of traversing each point in the map) and the cost of changing direction. When changing direction the vehicle needs to ensure the position is correct. Typically a change in direction is to drive around an obstacle so if you change direction at an inappropriate time can have devastating consequences. Think about driving around a crater or a big boulder for example.
  The generated path is implemented in a way that the onboard software can follow the intended route. Typically paths are approximated with a polyline where the vehicle is commanded to drive in DV, a specific distance at a set heading (see the Proposed Metrics section for more details on how to calculate DV on the calculated paths following the Feng and Pavlidis approach^25^).
3. **Analyze the rover’s mobility** behaviour along the selected path using the Hyperdrive simulator^26^. In order to analyze and test how the vehicle can behave on the terrain, simulators for each mission have been developed. By using a 3D terrain mesh generated from the images taken by the rover, Hyperdrive provides the status of the rover flight software including simulated telemetry such as rover attitude (roll, pitch, yaw) and rover position. It can help in setting safety parameters but it mostly rests on the drivers’ experience to balance risks by setting appropriate measures to maximize science return without risking the mission.
4. **Sequencing** rover commands are assembled in sequences that are developed during the course of an (Earth) day. A team of engineers analyzes first, the rover telemetry to know the state of the vehicle and the success of previous activities. If the next activity includes a move of the rover to a new location, the path and final position of the rover are negotiated with the rest of the team. Then, rover drivers define the resources needed for each planned activity: time slot for execution on Mars, power requirements, and volume of data generated by them.
  Sequences are then refined by including all ancillary commands that provide appropriate setup, safety parameters, as well as details regarding images to be taken after the mobility activity is completed. The sequences are presented to the team and verified parameter by parameter before displaying an animation of the movement. Once approved, all sequences are delivered to the Sequence Integration Engineer (SIE) who will merge and verify that there are no interactions between activities that would violate procedures, flight software limitations, or resource usage. Around 15:00 the team has a final review of all activities, procedures, and possible errors discovered while integrating all sequences. All sequences are now ready to be transmitted to the rover which will execute them autonomously.
  Starting with a draft in block a), all the way to the final version in block c), the sequencing tasks are as follows:
  - Navigation Mode: based on terrain assessment, determine the most appropriate navigation mode in each section of the drive path. The reader can appreciate the correlation between the terrain traversability (step 1) and the navigation modes. There are the following modes defined for MER and Curiosity (for more information about Perseverance navigation modes please refer to Verma et al.^1^:
    Blind driving (BD): typically used on terrain without obstacles and hazards. No cameras are used to locate obstacles or measure vehicle slip.
    Visual Odometry (VO): compares images taken before and after a step, and onboard machine vision software corrects the rover position computed by dead-reckoning. The flight software can autonomously trigger VO under certain conditions such as if the Inertial Measurement Unit (IMU) senses that the rover is currently traversing steep terrain. VO allows the measurement of the vehicle slip and therefore provides a better estimate of the vehicle position. In this mode, the vehicle will take more time to cover the same distance over BD.
    Autonomous Navigation or Autonav (AN): is used in areas where large rocks or steep scarps are expected or in terrain beyond what is visible from the previous location. The rover software can be configured to use its own cameras to locate obstacles, and steep or rough terrain and modify its own path to go around these hazards. In case the routing software cannot find a safe path the vehicle stops and signals a fault condition which will be seen by the operators on Earth the following day. This driving modality reduces the vehicle speed even more than VO.
    VO with AN: in the unlikely scenario that the rover needs to move on high-slip terrain where obstacles are present, it is necessary to activate both VO and AN. The effective drive rate is very low.
  - Set FSW safety parameters: to prevent the vehicle motion in unsafe situations. Limits can be set on tilt, yaw, slip, VO, *keep in zone* (KIZ, areas where the rover is allowed to move) and *keeps out zone* (KOZ, areas where the rover is not allowed to enter).
  - Finalize sequence: all setup commands, safety parameters, and drive commands are completely defined and verified by means of simulators and scripts that cross-check the sequence.
  - Post-drive imaging: after each mobility activity, it is necessary to capture images of the terrain surrounding the rover. Since there are limited resources, the drivers need to pick the most appropriate combination of cameras, filters, image compression level, and priority so that they can all fit in the expected downlink volume of data. Typically, front and rear HAZCAM (stereo pairs of 1-megapixel monochrome cameras with 120$^o$ field of view installed on the rover’s body) as well as a mosaic of 5 NAVCAM (1-megapixel monochrome stereo pair cameras mounted on the rover’s camera head with a 45$^o$ field of view and up to 50-80 meters of range) frames, are captured. If the following mobility activity requires it and if the downlink volume allows it, either PANCAM (on MER) or MASTACAM (on Curiosity) mosaic of several frames is also included.

## Path planning algorithms

The most common terrain discretization used for grid-based path planning algorithms is a regular 2D-grid with blocked and unblocked square cells^27^. For this kind of grids, a valid path is that starting from the initial node reaches the goal node without crossing a blocked cell. For real applications, it is obvious that the binary values for grids are not enough and the classical algorithms should be extended in order to handle cells with an associate traversal cost (cell cost) that reflects the difficulty of navigating that area of the terrain (as described in the previous section). These kinds of abstractions are called cost or traversability maps. Next, we briefly describe the features of the two algorithms used in this article, which work over cost maps.

### Field D*

Field D* belongs to the family of algorithms (D* Lite^28^) that use interpolation to produce better value functions for discrete samples over a continuous state space. The innovation in Field D* algorithm is a method for computing the cheapest path of each grid node *s* to the goal, given the path costs of its neighboring nodes ($ns_{i}$). This value is traditionally computed as the minimum cost of traversing the edge between *s* and any of its neighboring nodes $ns_{i}$ plus the path cost of the chosen *ns* to the goal. But this computation only allows straight lines from *s* to one of the $ns_{i}$. However, Field D* allows a straight-line trajectory from node *s* to any point on the boundary of its grid cell of the $ns_{i}$. Since computing all the boundary nodes is infinite, it provides an approximation for each boundary point by using linear interpolation. Then, the cost of an edge that resides on the boundary of two grid cells is defined as the minimum of the traversal costs of each of the two cells.

The search process continues until the goal is found. Then, the path is extracted by starting at the initial position and iteratively computing the cell boundary point to move next. Although in some cases, as reported by Ferguson and Stentz^5^, the linear interpolation returns a bad approximation, in general results show the benefits of the algorithm.

### 3Dana

The 3D Advanced Navigation Algorithm (3Dana) is a path-planning algorithm designed to deal with realistic surface scenarios. In that sense, it can integrate during the search the DTM information in combination with a cost map, in order to try to generate safer routes, avoiding potentially dangerous areas and excessive slopes, while keeping the path length and heading changes as low as possible. The search algorithm is based on A* with vertex re-expansion and integrates the Line of Sight check during search such as Theta* does. However, the line of sight check implemented in 3Dana provides the exact length for a segment that crosses a cell, while Theta* and other derived works only provide estimations at that point^29^. This allows the algorithm to retrieve the real path cost for a given region in a cost map, which is the result of multiplying the segment length that crosses a cell by the cell cost. Another advantage is that, with this line of sight, it supports DTM information to perform any-angle paths using a linear interpolation method to obtain elevation for non-vertex points, allowing one to obtain the path length in non-flat surfaces. Also, using the DTM and the interpolation it is able to compute terrain slopes at a given point. In this regard, 3Dana accepts a maximum slope constraint, discarding those paths that are required to overcome the maximum slope value.

For the search heuristic, 3Dana takes into consideration both, the distance to the goal (which includes the altitude using a variation of the Euclidean distance) and the heading change required. The last one is inherited from the S-Theta* algorithm and guides the search considering not only the path length but also the current heading of the robot. This helps minimize the total turns required in the generated path. In fact, it is possible to modify the weight of this heuristic, giving more or less relevance to the heading changes according to the operational requirements.

## Proposed metrics

Classical metrics for path planning algorithms are objective criteria: total distance traveled, search run-time, and number of expanded nodes. The first criterion assumes that the optimal path is the shortest. Although our intuition tells us that it is probably the optimal solution the best one, it might in fact incur additional risks and costs. While a windy road might be shorter, the longer freeway might be faster, easier to follow, and less risky. While there are no roads on Mars, similar concepts apply there as well. Following a windy path is much more complex to implement and every turn needs to be carefully traced by the rover to avoid surrounding hazards or obstacles.

The other classical metrics try to measure the computational complexity and the memory usage of the algorithm. Especially for embedded systems or planetary rovers where processing power and storage are at a premium, those parameters are critical but when algorithms are run as part of strategic route planning these are seldom limiting factors as ground support equipment can be tailored for this task and real-time response is not required.

So, in the context of path planning, what is really important?

In order to answer that question, let’s take a closer look at some aspects of the criteria used to select rover paths. In general, we can say that each path is evaluated in terms of 4 criteria:

- Total length.
- Terrain traverse cost.
- Accumulated drive time.
- Ease of implementation.

Next, we describe in detail each criterion or *metric*.

The **total length** is specified as a set of straight line segments connecting the starting location through a series of intermediate WPs and finally to the goal location. In this case, the path length is computed as the sum of the Euclidean length of each straight-line segment. Curvilinear paths are approximated with a polyline where the vehicle is commanded to drive in DV, a specific distance at a set heading. Each drive vector (DV) is implemented as a set of commands. Reducing the number of DV lowers the activity complexity and risk. In order to approximate the paths, we have adapted the vision algorithm by Feng and Pavlidis^25^ that extracts polygonal outlines of objects in a picture, to generate polylines of the paths generated by the path-planning algorithms.

The control software on board the Mars rovers uses feedback measured after each step is taken. Due to hardware limitations, steps can be as short as 50cm (for MER class vehicles) or as long as 1m (for MSL class vehicles). As a result, it is much easier to have the vehicle follow a path defined as a set of line segments. Each segment will require only a handful of commands while a curved, sinuous path would require many more drive and control commands which would increase the risk of commanding errors. We have to keep in mind that each drive path is designed on Earth and uplinked and executed on Mars in complete autonomy, so the simpler its design, the lower the risk of an anomaly.

Each DV has a tolerance band which depends on its length, so a 1 m segment will have a much smaller tolerance band than a 100 meter. In our case the tolerance is 10%. The ground surface composition and the vehicle roll while following a straight line segment are the two most important factors affecting the deviation from the designed path. All areas that are deemed traversable ensure that the actual deviation is less than 10%.

The **terrain traverse cost** can be defined as the time it takes for the rover to follow the path. Total traverse duration is computed as the sum of traverse time for each cell along the path. Although traversability cost is proportional to traverse time, other factors need to be taken into account such as the altitude since 3Dana uses both, cost and altitude, and it needs to be considered in the computation time.

During Winter less energy is available for driving, temperatures are colder, daylight is shorter and therefore it will take more Sols to drive the same distance. Also, terrain viewshed affects how long it takes for the rover to traverse a specific area. Since the HiRISE camera cannot resolve and detect all mobility obstacles, areas deemed free of obstacles that are occluded by surface topography at the time the rover begins the actual drive will be traversed using AutoNav, a “drive rate” or driving modality (i.e. distance per unit of time) that takes a significantly longer time. The inverse of the drive rate provides the traverse time corresponding to each drive rate.

Each class of Martian rovers has specific drive rates for each software module as their implementation and on-board processors have different performances. To correctly model the traverse duration, the path is traced along the cost map from the starting location. For each traversed cell the cost is converted into drive rate and the corresponding drive time is accumulated.

**Accumulated drive time** is converted into “Drive Sols” by considering that for every Sol on Mars the allocation of energy available to driving changes according to the Season. In Winter more energy is devoted to heating the drive actuators than in Summer and therefore there is less available energy for mobility activities. For solar-powered vehicles like MER, in Winter the Sun is also lower on the horizon further reducing the amount of available energy for mobility activities. While tracing the path, a Drive Sol is counted when the daily energy allocation for mobility is depleted, and at this point, a new starting position is established. This metric accounts for dynamic energy management in response to seasonal variations and operational demands, ensuring clarity in its computation.

Finally, **ease of implementation** is expressed in terms of the number of vehicle commands to execute the specific path. Each drive Sol is a pre-programmed sequence of commands that implement a finite set of DV, a distance to travel in a specific direction or toward a set location. Each DV has associated motion commands as well as safety parameters. Sequence complexity is therefore directly related to the number of DV. The lower number of DV as well as average heading changes are desirable as they reduce the sequence complexity and risk. Longer DV instead would simplify the number and nature of commands in a sequence. Errors in the execution of heading changes can have more severe consequences when the heading is larger. As a result, the average heading change is more related to sequence risk rather than complexity.

Ease of implementation is therefore summarized as:

- Number of DV.
- Average length of DV.
- Average change of heading between DV.

## Experiments

In this section, we will show the values of the metrics presented in the previous sections for the two missions operated by JPL on the Martian surface. We have run Field D* (i.e. the path planning algorithm used at JPL) and 3Dana (with different parameters for path search configuration). We have used a 3Dana configuration in which the DTM is not considered, just the cost map (since Field D* cannot handle the DTM information directly). Also, we have employed different weights (presented as ’*a*’ in the tables and figures) for the heuristic that guides the heading changes during the search. In that sense, four possibilities have been taken into consideration: 0, 0.25, 0.5 and 1.

3Dana $a$ $=$ $0$ or just 3Dana is a configuration in which the heading changes are not considered during path search, while $a$ $=$ $1$ means that the relevance of the heading changes heuristic is similar to the path length one. In that sense, as bigger is *a*, more impact has the heading changes during the search and it is expected that lower turns are produced in the path. Experimentally, higher values than 1 do not provide better results and, sometimes, return paths with more heading changes than smaller values. Using values higher than 1 implies that the algorithm tends to avoid rotations and move forward even when it implies moving away from the destination. Therefore, corrections in the path were required, reducing the performance of the solution. This issue is presented in^7^ along with the alpha formulation.

Path planning algorithms were executed on a 2014 Mac Pro, 12 Core, 64GB RAM with cost maps stored on SSDs. Run time was about 5 seconds for Field D* and about 32 seconds for 3Dana. In both cases path planning time can be considered quite acceptable.

In the next two examples, we show how classical metrics might not fully capture the properties of different path-planning algorithms. We want to remark that the WPs used for the path search are the ones traversed by the Mars rovers. Particularly, the Field D* path shows a similar path to the one followed by the rover (as it was the algorithm used during the operation), meanwhile, 3Dana presents alternative paths, which, as can be seen, are very similar depending on the configuration used. It is important to mention that both algorithms work with the same cost function, i.e., they only considered the distance traveled and the terrain cost to compute the path quality during the search. In the case of 3Dana, moreover, the configuration of the $\alpha$ value allows us to consider the heading change as part of the heuristic function.

**Figure 2:**
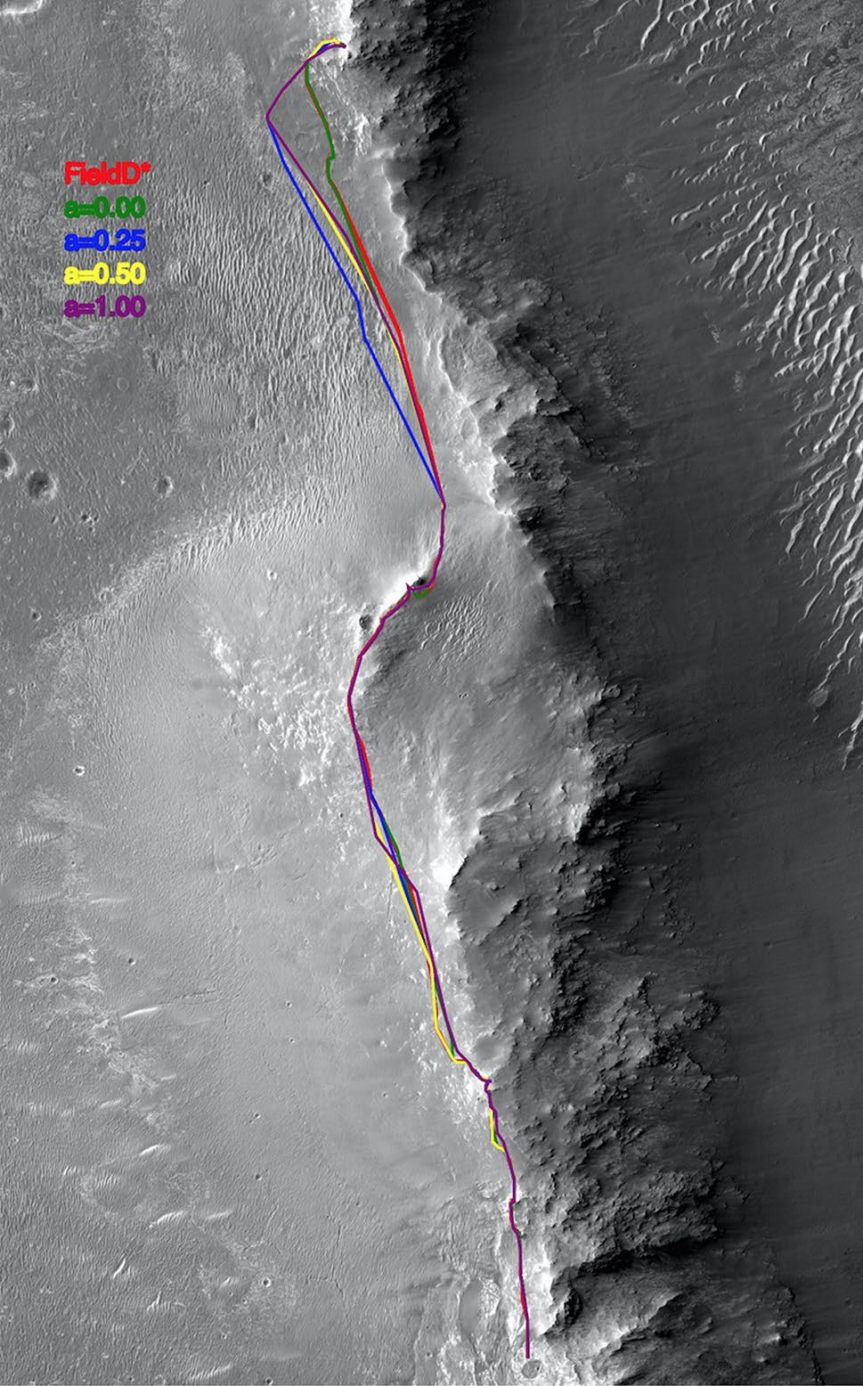
Solutions for the Opportunity map example.

**Table 1:** Results of the two algorithms under different metrics for Opportunity.

Path name	Path length [m]	DVs	Average DV length [m]	Average heading change [deg]	Duration [Minutes]	Duration [Sols]
3Dana	2244.09	93	24.13	27.12	4468.60	109
3Dana a = 0.25	2269.71	87	26.09	24.27	4514.51	110
3Dana a = 0.50	2274.31	78	29.16	27.66	4516.86	110
3Dana a = 1.00	2251.62	74	30.43	23.31	4802.23	116
Field D*	2008.98	712	2.83	70.13	4326.82	106

The *first example* shows Rover Opportunity path planning. In July 2014 the MER project wanted to verify that before the upcoming Martian Winter (October 2015) the Opportunity vehicle could reach a smectite-rich area on the western rim of Endeavour Crater in Meridiani Planum. This path was actually executed on Mars from Sol 3710 (July 1, 2014) to Sol 3967 (March 22, 2015). The path was completed over the course of 257 Sols, about 9 Earth months. The terrain along the route was a combination of smooth terrain, dune fields, medium-sized rock areas, several scarps, and bedrock with significant elevation changes and terrain slopes in the range of 0-25 degrees. While not particularly challenging it still had some potential embedding events and areas where there with some obstacles-dense areas that needed careful planning.

The size of the map is 4854 x 5000 cells, where one cell is 1.01 x 1.01 meters. We have employed this cell size because it closely corresponds to the dimensions of the rover, ensuring a snug fit within each cell. Subsequently, obstacles are expanded or “dilated” to match these cell dimensions. The utilization of larger cells can introduce precision limitations when assessing potential hazards, as they might not fully encompass the intricate details of the rover’s immediate environment. Conversely, opting for smaller cells could lead to excessive grid precision that surpasses the operational requirements, taking into account the actual size of the rover. Striking the appropriate balance between granularity and practicality is a crucial consideration in the path-planning process. Figure 2 shows the different solutions on the Martian surface.

Table 1 reports for each of the evaluated path planning algorithms the total path length in meters as well as the projected path duration, that is the expected time for the rover to traverse such path. Duration is given both in minutes and Sols.

Obviously minimizing path length and duration are desirable features in order to minimize wear and tear on the vehicle and to maximize time that can be devoted to scientific observations. The table also reports the total number and average length of DV as well as the average heading change. Minimizing the number of DV and heading change values helps in reducing operations complexity, risk, and cost. As each path is computed as a series of cell locations, DV are defined as sets of successive cells that do not deviate from a straight line by more than 10 percent of the vector length. These were computed by implementing polygonal approximation algorithms^25^ adapted for this task.

In this example, Field D* minimizes path duration but the other 3Dana configurations have values that are not too dissimilar. The number of DV and heading changes are substantially different with Field D*, about one order of magnitude larger than the others. This difference can have a dramatic impact on the risk and complexity of rover operations.

In the *second example* the Curiosity Rover landed at Gale Crater on August 6 (UTC) 2012 at a location christened Bradbury Landing in honor of the author who at that time had recently passed away. While the primary goal of the mission was to reach the base of Mt. Sharp as quickly as possible there was a request to first visit Yellowknife Bay, an area about half a kilometre East of the landing site where orbital imagery showed transitions between several terrain types.

Before heading South West to an area named The Kimberley the MSL project wanted to quantify the impact on mission duration caused by this deviation. The terrain was overall a mixture of smooth but rocky terrain with occasional Aeolian drifts and sand dunes, exposed bedrock and hard and sharply pointed rocks called ventifacts, rocks that are shaped by the incessant action of wind and sand erosion. This terrain needed to be swiftly traversed to reach science targets that were several kilometers apart.

**Figure 3:**
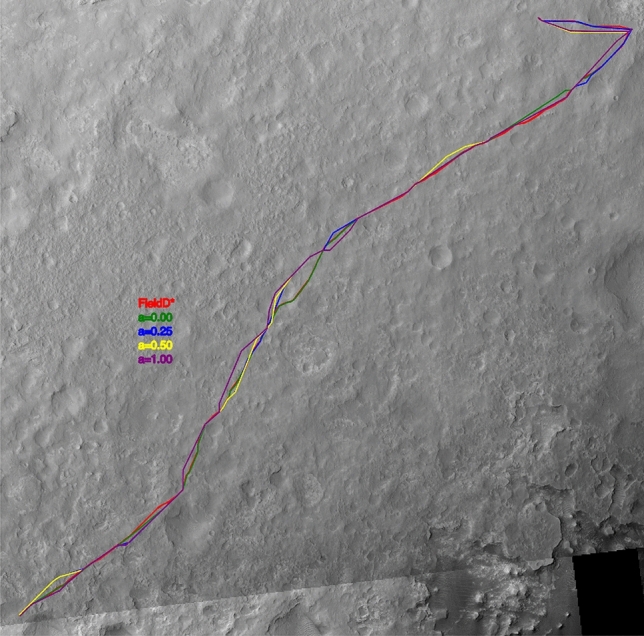
Solutions for the Curiosity map example.

The size of the map is 30016 x 46253 cells with 1 meter spacing. In this case, we are focusing on a small part of the DTM with nearly 16km$^2$, where Curiosity is located. The generated paths can be graphically seen in Figure 3.

In this second case, the difference between the various path planning algorithms is quite remarkable as Table 2 shows. While the difference in total path length and projected duration is negligible, the number and average length of DV spans almost two orders of magnitude. This difference is quite remarkable and its impact on operational risk can have a significant impact on mission outcome and science return.

**Table 2:** Results of the two algorithms under different metrics for Curiosity.

Name	Path length [m]	DVs	Aver. DV length [m]	Aver. heading change [deg]	Duration [Minutes]	Duration [Sols]
3Dana a = 0.00	3487.63	48	72.66	17.82	3625.73	76
3Dana a = 0.25	3517.63	41	85.80	21.88	3654.88	77
3Dana a = 0.50	3531.86	38	92.94	27.59	3656.94	77
3Dana a = 1.00	3526.89	35	100.77	28.89	3669.34	77
Field D*	3406.27	789	4.31	78.50	3617.67	76

Then, from both examples, we can conclude that while there’s no significant difference in duration between the various path planners tested, it is important to note that 3Dana will significantly reduce the number of DVs, making the design of each drive Sol path much easier to implement.

In a typical MSL sequence, we have a set of about 23 setup commands and 7 teardown commands. Each DV requires about 8 commands. Even just assuming the risk is uniformly distributed among all types of commands, each DV has a significant portion of each Sol drive. The reality is that most of the 30 (23+7) setup/teardown commands have mostly restrictive roles while each DV is an active command that can put the vehicle and mission at risk.

The actual drive time for MSL (that is, how much time it took to drive the path) was 185 drive sols (almost double as shown in Table 2). The difference between the actual and the predicted time is due to the fact that various science targets were introduced by the scientists along the path, and then new drives were necessary for small and precise rover placement after the new targets were introduced.

To conclude, the great differences in DVs between 3Dana and Field D* are related to how both algorithms perform the search. In 3Dana, heading changes are only allowed at the edges of the nodes. Moreover, 3Dana implements a heuristic to consider heading changes during the search phase, effectively reducing the heading changes. Meanwhile, in Field D* the path is not restricted to traverse through node edges. The search procedure implements an interpolation along edges, enabling heading changes not only at edges but also in between them. The interpolation is performed based on the terrain’s properties, in this case, the elevation. Therefore, Field D* only considers the traversal cost to decide when and where to perform a rotation. When applied to such complex terrains, the algorithm tries to avoid even small elevation changes, resulting in more rotations in the generated path and thus, more DVs required to execute the route.

## Discussion

This paper ignites a crucial debate within the research community, challenging conventional metrics used to evaluate path-planning algorithms. These algorithms are instrumental in determining optimal routes between start and end points while navigating obstacles and hazardous terrains. Commonly, assessments rely on metrics such as path length, runtime, and node expansions.

Our aim is not to diminish the importance of classical metrics but to broaden our perspective, delving into the intricacies of path planning in unstructured environments, especially in planetary exploration. It is imperative to stress that a path planner prioritizing minimal turns is undeniably preferable to a planner that, while minimizing traverse cost, introduces a plethora of turns. This preference arises from the fact that simplicity in path execution significantly reduces operational risk. The analogy of a shorter, winding road versus a longer, smoother route underscores the significance of minimizing turns, as it can substantially impact mission success.

In response to these complexities, we introduce a quartet of evaluation metrics. They transcend classical assessments and delve into multifaceted considerations defining path quality for autonomous rovers. Moreover, it is important to take note of the DV: as each DV is implemented as a set of commands, reducing the number of DV lowers the activity complexity and risk of errors both for human operators that must program the routes and communication related issues. In this regard, reducing the number of DVs also reduces the required bandwidth required for sending the planning to the explorer.

This paper extends an open invitation to the research community to engage in robust debate on metrics that genuinely matter in the realm of path planning. By collectively redefining our understanding of optimal paths, we aim to shape the future of planetary exploration and autonomous ground vehicle navigation in unstructured environments.

## Conclusions

Effective path-planning is pivotal to the operational success of Mars rovers, demanding careful consideration of scientific objectives, terrain intricacies, energy constraints, and safety imperatives. While conventional metrics like path length, runtime, and node expansion counts have been standard in path evaluation, we embarked on a critical inquiry into their suitability in the face of formidable and partially known terrains.

Drivers on Mars frequently use path-planning tools to plan the route that the rovers need to follow for several weeks or months. But the optimal route, that is, the best to follow, is not always the shorter one. Based on the experience of JPL rover drivers, the tools they use, and high-resolution Mars maps, we propose new metrics to capture most of the criteria assessed by rover drivers during daily operations: Terrain Traverse Cost, Accumulated Drive Time, and Ease of Implementation. The objective of these metrics is to introduce a better assessment of path-planning algorithms for reducing activity complexity and risk when operating real robots.

## Data Availability

The maps used can be downloaded directly from HiRISE. For the Opportunity test, ESP_058208_1775 can be downloaded at: https://www.uahirise.org/ESP_058208_1775 and for the Curiosity test, we have used ESP_030168_1755, which can be found at: https://www.uahirise.org/ESP_030168_1755. The code used during the current study is available in the GitHub repository: https://github.com/munozp/up2ta/tree/master/src/PathPlanning.
